# World Input-Output Network

**DOI:** 10.1371/journal.pone.0134025

**Published:** 2015-07-29

**Authors:** Federica Cerina, Zhen Zhu, Alessandro Chessa, Massimo Riccaboni

**Affiliations:** 1 Linkalab, Complex Systems Computational Laboratory, Cagliari, Italy; 2 Department of Physics, Università degli Studi di Cagliari, Cagliari, Italy; 3 IMT Institute for Advanced Studies Lucca, Lucca, Italy; 4 Department of Managerial Economics, Strategy and Innovation, Katholieke Universiteit Leuven, Leuven, Belgium; Universidad Rey Juan Carlos, SPAIN

## Abstract

Production systems, traditionally analyzed as almost independent national systems, are increasingly connected on a global scale. Only recently becoming available, the World Input-Output Database (WIOD) is one of the first efforts to construct the global multi-regional input-output (GMRIO) tables. By viewing the world input-output system as an interdependent network where the nodes are the individual industries in different economies and the edges are the monetary goods flows between industries, we analyze respectively the global, regional, and local network properties of the so-called world input-output network (WION) and document its evolution over time. At global level, we find that the industries are highly but asymmetrically connected, which implies that micro shocks can lead to macro fluctuations. At regional level, we find that the world production is still operated nationally or at most regionally as the communities detected are either individual economies or geographically well defined regions. Finally, at local level, for each industry we compare the network-based measures with the traditional methods of backward linkages. We find that the network-based measures such as PageRank centrality and community coreness measure can give valuable insights into identifying the key industries.

## Introduction

Ever since Leontief [1] formalized its structure, the input-output table has been used extensively by economists, environmentalists, and policy makers alike. By keeping track of the inter-industrial relationships, the input-output table offers a reasonably accurate measurement of the response of any given economy in the face of external shocks or policy interventions. As the global economy becomes increasingly integrated, an isolated view based on the national input-output table is no longer sufficient to assess an individual economy’s strength and weakness, not to mention finding solutions to global challenges such as climate change and financial crises. Hence, a global multi-regional input-output (GMRIO) framework is needed to draw a high-resolution representation of the global economy [2]. In practice, however, due to the expensive process of collecting data and the variety of classifications used by different agencies, for a long time, the input-output tables have only been available for a limited number of countries and for discontinuous years. Fortunately, the fully-fledged GMRIO databases started to become available in recent years. Tukker and Dietzenbacher [3] have summarized the recent development of the GMRIO databases.

Unlike the national input-output table where exports and imports are aggregated and appended to final demand and country-specific value added respectively, for each individual economy, the GMRIO table splits its exports into intermediate use and final use in every foreign economy and also traces its imports back to the industry origins in every foreign economy. As a result, the inter-industrial relationships in the GMRIO table are recorded not only within the same economy but also across economies.

The availability of the GMRIO databases was soon followed by a wave of empirical studies of topics ranging from global value chains and trade fragmentation in economics [4–7] to global environmental accounting in ecology and resources management [8–10]. However, to the best of our knowledge, our paper is the first attempt to systematically explore the GMRIO tables from a network perspective. In contrast, some previous studies only consider the input-output network at the national level [11–13] while others only investigate a limited number of countries or years of the GMRIO tables with simplified network methods [14, 15].

Complex networks can be used to characterize a series of different systems where multiple agents (nodes) interact with each other through their relationships (edges) [16, 17]. This approach has provided new insights in understanding complex phenomena in various fields ranging from biology [18] to economics [19–21] and finance [22]. From a network perspective, we consider the GMRIO system as a world input-output network (WION), where the nodes are the individual industries in different economies and the edges are the monetary goods flows between industries. The direction of the flows goes from the seller industry to the buyer industry. They are monetary because they are denoted in current US dollars. In a broad sense, our work is related to the fast-growing literature of the international trade networks, where countries are represented by nodes and trade relationships are represented by edges [23–31]. However, there are two key differences between the WION and the trade networks. First, the WION is based on industries. Not only the cross-country industrial relationships, but also the domestic ones can be studied. Second, the WION focuses on intermediate transactions (i.e., products used for further production) while the trade networks aggregate both final (i.e., products used by final consumers) and intermediate trade flows. As the international trade is increasingly characterized by intermediate transactions and hence by global value chains [32], the WION certainly becomes a more appropriate methodology than the trade networks to study the world production system and the comparative advantages among countries and industries.

As a specific network, the WION has the following features: 1) It is directed and weighted, i.e., an industry can act as both a seller and a buyer at the same time and the monetary goods flows between industries vary considerably in volume; 2) It is much denser within the same economy than across economies, i.e., despite the continuously integrated global economy, most economic transactions still happen within the country border. In contrast, due to the low-digit industry classification, the input-output networks at the national level are typically dense and complete [11]; 3) It is with strong self-loops, i.e., an industry can acquire a significant amount of inputs from itself, which is again due to the aggregated industry classification.

Taking into account the features above, we explore the WION by analyzing its global, regional, and local network properties respectively. This paper makes some significant contributions to the literature of input-output economics. First, unlike Carvalho [14] who uses only a single-year (2006) GMRIO table and considers it as an unweighted network, we quantify the global network properties of the WION over time and take into account its edge weights and directedness. We find that the increasingly integrated production chains tend to make the WION more clustered and more assortative. We also find that the industries are both highly connected, as captured by the degree distribution, and asymmetrically connected, as captured by the strength distribution, which implies that local shocks can propagate through the world economy and lead to a sizable global fluctuation. Second, at regional level, we study its subgraph structure and dynamics by using community detection techniques. We find that the world production is still operated nationally or at most regionally as the communities detected are either individual economies or geographically well defined regions. In particular, we detect the emergence of a large European community led by Germany, which is most likely the result of the production activities outsourced in recent years from Germany to Central and Eastern European countries with lower labor costs and growing demand. Finally, we quantify some local network properties of the WION and compare them with the traditional methods of backward linkages. We find that the network-based measures such as PageRank centrality and community coreness measure can give valuable insights into identifying the key industries.

The rest of the paper is structured as two sections. The following section first describes how we construct the WION and then studies its network properties at global, regional, and local levels respectively. The last section discusses some policy implications of our results and concludes the paper.

## Methods and Results

### Construction of the WION

Our construction of the WION is based on the World Input-Output Database (WIOD) [33]. At the time of writing, the WIOD input-output tables cover 35 industries for each of the 40 economies (27 EU countries and 13 major economies in other regions) plus the rest of the world (RoW) and the years from 1995 to 2011 (S1 Table and S2 Table have the lists of countries and industries covered in the WIOD.). For each year, there is a harmonized global level input-output table recording the input-output relationships between any pair of industries in any pair of economies. The relationship can also be an industry to itself and within the same economy. The numbers in the WIOD are in current basic (producers’) prices and are expressed in millions of US dollars. Table 1 shows an example of a GMRIO table with two economies and two industries. The 4 × 4 inter-industry table is called the transactions matrix and is often denoted by **Z**. The rows of **Z** record the distributions of the industry outputs throughout the two economies while the columns of **Z** record the composition of inputs required by each industry. Notice that in this example all the industries buy inputs from themselves, which is often observed in real data. Besides intermediate industry use, the remaining outputs are absorbed by the additional columns of final demand, which includes household consumption, government expenditure, and so forth (Here we only show the aggregated final demand for the two economies). Similarly, production necessitates not only inter-industry transactions but also labor, management, depreciation of capital, and taxes, which are summarized as the additional row of value added. The final demand matrix is often denoted by **F** and the value added vector is often denoted by **v**. Finally, the last row and the last column record the total industry outputs and its vector is denoted by **x**.

**Table 1:** A hypothetical two-economy-two-industry GMRIO table. The 4 × 4 inter-industry transactions matrix records outputs selling in its rows and inputs buying in its columns. The additional columns are the final demand and the additional row is the value added. Finally, the last column and the last row record the total industry outputs. The numbers are made up in such a way that Economy 2 is much larger than Economy 1 in terms of industry outputs. However, as shown below, the Rasmussen method of backward linkages will consider industries in Economy 1 more important than the ones in Economy 2. Hence, we use the Laumas method of backward linkages instead to identify the key industries in the WIOD.

		Buyer Industry						
		Economy 1		Economy 2		Final Demand		
Seller Industry		Industry 1	Industry 2	Industry 1	Industry 2	Economy 1	Economy 2	Total Output
Economy 1	Industry 1	5	10	20	10	45	10	100
	Industry 2	10	5	10	20	50	5	100
Economy 2	Industry 1	30	15	800	500	5	8650	10000
	Industry 2	35	30	1000	1000	25	7910	10000
Value Added		20	40	8170	8470			
Total Output		100	100	10000	10000			

As mentioned above, the complex networks approach has been widely used in economics and finance in recent years. Designed to keep track of the inter-industrial relationships, the input-output system is an ideal test bed for network science. Particularly the GMRIO system can be viewed as an interdependent complex network, i.e., the WION, where the nodes are the individual industries in different economies and the edges are the flows between industries.

**Fig 1:**
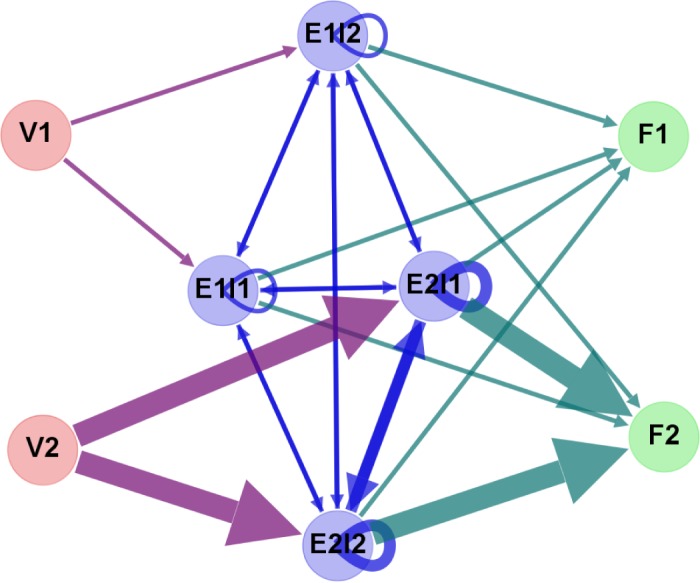
A hypothetical two-economy-two-industry WION. This is a topological view of Table 1. The blue nodes are the individual industries. The label “ExIy” should read “Industry y in Econoomy x”. The red nodes are the value added sources from the two economies, whereas the green nodes are the final demand destinations in the two economies. The label “Vx” should read “Value Added from Economy x”, whereas the label “Fx” should read “Final Demand in Economy x”. The edges are with arrows indicating the directions of the monetary goods flows and with varying widths indicating the magnitudes of the flows. Finally, because we are only concerned with the inter-industrial input-output relationships, when formulating the WION, we focus our attention on the network among the blue nodes.

Fig 1 provides a topological view of Table 1. The blue nodes are the individual industries. The red nodes are the value added sources from the two economies, whereas the green nodes are the final demand destinations in the two economies. The edges are with arrows indicating the directions of the monetary goods flows (Strictly speaking, the flows from the red nodes to the blue nodes are not goods but primary inputs in nature.) and with varying widths indicating the magnitudes of the flows. Finally, because we are only concerned with the inter-industrial input-output relationships, when formulating the WION, we focus our attention on the network among the blue nodes. In other words, we construct the WION using **Z** as its weighted adjacency matrix.

**Fig 2:**
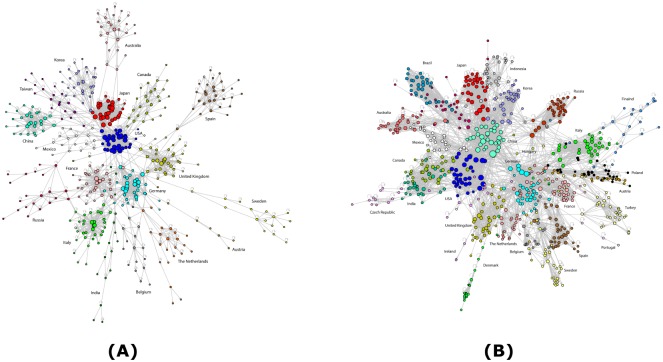
The WION in 1995 and 2011. Each node represents a certain industry in a certain economy. The size of the node is proportional to its total degree (number of edges). The edges are directed and only those with strength greater than 1000 millions of US dollars are present. Finally, different colors represent different economies.

The filtered version of the constructed WION in 1995 (Panel (A)) and 2011 (Panel (B)) are shown in Fig 2. Each node represents a certain industry in a certain economy. The size of the node is proportional to its total degree. The edges are directed and only those with strength greater than one billion US dollars are present. Finally, different colors represent different economies. Clearly the WION has become denser over time and some countries like China have moved to the core of the network.

In the following subsections, we quantify the network properties of the WION at global, regional, and local levels respectively and document its evolution over time.

### Global Analysis of the WION

**Fig 3:**
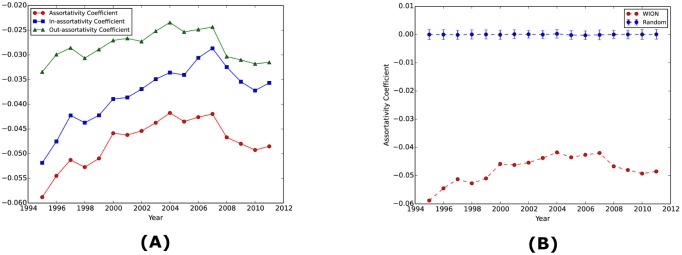
Assortativity of the WION over time. In Panel (A), from top to bottom, we show the over time out-degree assortativity, in-degree assortativity, and total-degree assortativity, respectively. Panel (B) compares the total-degree assortativity calculated from the original WION with that calculated from its random counterpart which preserves the degree distribution of the original network. For each year the random counterpart is simulated for 50 times and the 95% confidence interval is shown. Therefore, even with small magnitude, the assortativity coefficients are nevertheless statistically significant.

**Fig 4:**
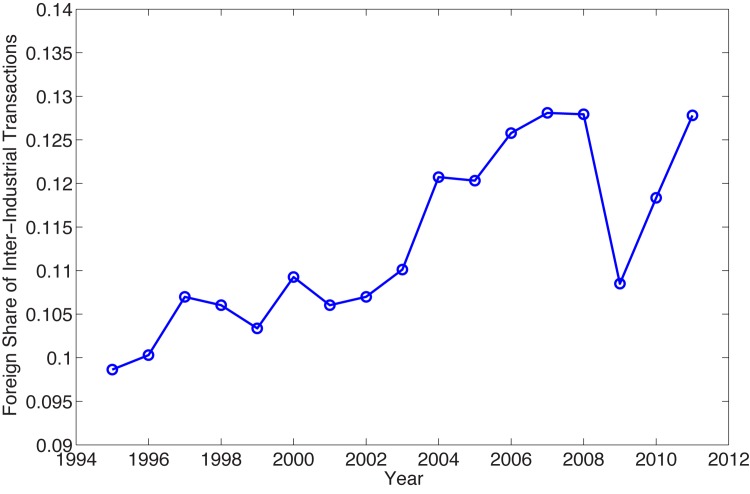
Foreign Share of the Intermediate Transactions. We calculate the foreign share of the transactions matrix **Z** over time. We calculate the percentage of inputs from foreign origins (or equivalently, the percentage of outputs to foreign destinations) of the transactions matrix **Z** of the 40 WIOD economies. The same hump-shaped behavior over time is observed here as in assortativity and clustering coefficient.

The global network properties we quantify for the WION include assortativity, clustering coefficient, and degree and strength distributions. Because the WION is directed, we can calculate the assortativity coefficient in three ways, namely, in-degree assortativity, out-degree assortativity, and total-degree assortativity. As shown in Panel (A) of Fig 3, they all behave similarly over time. First, they have all been negative throughout the whole period. Since assortativity measures the tendencies of nodes to connect with other nodes that have similar (or dissimilar) degrees as themselves, a negative coefficient means that dissimilar nodes are more likely to be connected. In the case of the WION, all the coefficients are of very small magnitude less than 0.06, but with statistical significance as shown in Panel (B) of Fig 3. Notice that when calculating the assortativity for in-degree, out-degree, and total-degree, respectively, we consider the nodes as the neighbors of a given node if they are connected with the given node by only incoming edges, by only outgoing edges, and by either incoming or outgoing edges, respectively. In contrast, Carvalho [14] defines the neighborhood solely on the basis of the incoming edges and finds a positive assortative relationship. One possible explanation of the negativity is that high-degree industries such as construction often take inputs (or supply outputs) from (or to) low-degree industries such as transport services. Moreover, the spatial constraints (each node has only few neighboring nodes in the same country) introduce degree-degree anticorrelations (disassortativity) since high degree sectors are in different countries and the probability to connect decays with distance [34]. Second, all the coefficients show an increasing trend before 2007 and a significant decline after 2007. The behavior of the assortativity measures seems to be correlated with the trend of the foreign share in the inter-industrial transactions over time (Fig 4). That is, we can calculate the foreign share of the intermediate transactions as the percentage of inputs from foreign origins (or equivalently, the percentage of outputs to foreign destinations) of the transactions matrix **Z** of the 40 WIOD economies. Same as observed in assortativity, the foreign share of **Z** has a steady growth (from 9.9% in 1995 to 12.8% in 2007) before 2007 and a sharp decrease after 2007 (While the most severely depressed domestic edges during 2008–2009 in terms of the magnitude of the reduced flows are mostly within USA, the top 3 most impacted foreign edges are all from the mining industry to the coke and fuel industry and are from Canada to USA, from Netherlands to Belgium, and from Mexico to USA, respectively.). The increase in the foreign share implies more interactions across economies and hence tends to make the WION less dissortative. The opposite happens when the foreign share goes down as a result of the global financial crisis. Third, we notice that the in-degree assortativity tends to be lower than the out-degree assortativity, but there is a tendency to close the gap between the two measures. We interpret this evidence as a clear signal of the integration of production chains, that is to say, both global buying and selling hubs have now a higher chance to be connected across borders.

**Fig 5:**
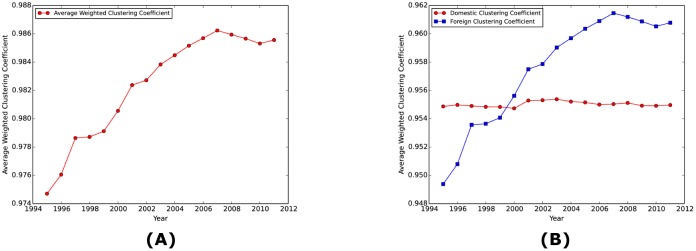
Clustering coefficient of the WION over time. Panel (A) shows the average weighted clustering coefficient of the WION over time. Panel (B) further decomposes the clustering coefficient into domestic clustering coefficient and foreign clustering coefficient. Clearly the behavior in Panel (A) is more explained by the foreign clustering coefficient.

The hump-shaped behavior is also observed in the clustering coefficient. Panel (A) of Fig 5 shows that the average weighted clustering coefficient of the WION has been steadily increasing but was followed by a decline since 2007. Again, a possible explanation is that the booming economy before 2007 introduced more interactions between industries, hence higher clustering coefficient, and the financial crisis after 2007 stifled the excessive relationships. Panel (B) further decomposes the clustering coefficient into domestic clustering coefficient and foreign clustering coefficient. Clearly the behavior in Panel (A) is more explained by the foreign clustering coefficient, which implies that the increasingly integrated production chains tend to make the WION more clustered.

We can also examine the global network properties of the WION by plotting its degree and strength distributions. Recall that the WION is based on the weighted adjacency matrix **Z**, i.e., the transactions matrix of the GMRIO table. Therefore, the degree and strength measures are different from the direct and total linkages used in the conventional input-output analysis, which are based on the technical coefficients matrix **A** and the Leontief inverse **L** respectively (see Subsection “Local Analysis of the WION” for the definitions). If we denote the regular binary adjacency matrix as **D**, where *D*_*ij*_ = *D*_*ji*_ = 1 if either *Z*_*ij*_ > 0 or *Z*_*ji*_ > 0, then we have the following definitions for a given node *i*: 1) In-degree: $D_{i}^{in}=\sum_{j\neq i}D_{ji}$; 2) Out-degree: $D_{i}^{out}=\sum_{j\neq i}D_{ij}$; 3) Total-degree: $D_{i}^{total}=D_{i}^{in}+D_{i}^{out}$; 4) In-strength: $S_{i}^{in}=\sum_{j\neq i}Z_{ji}$; 5) Out-strength: $S_{i}^{out}=\sum_{j\neq i}Z_{ij}$; 6) Total-strength: $S_{i}^{total}=S_{i}^{in}+S_{i}^{out}$.

**Fig 6:**
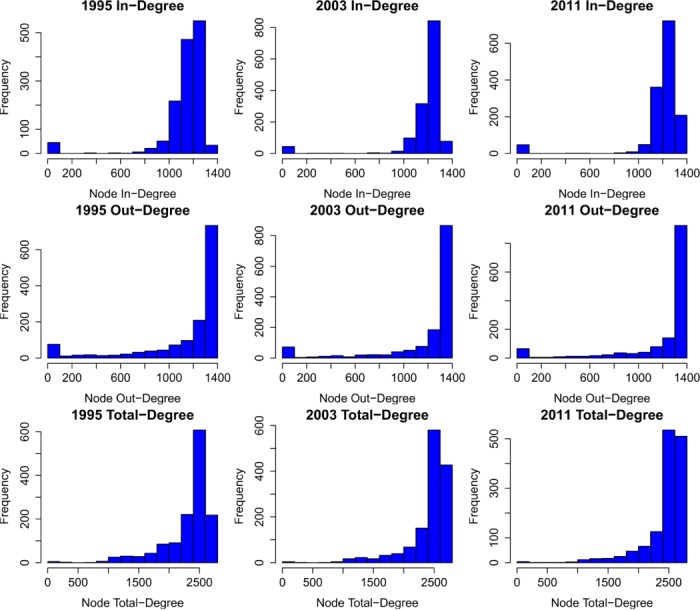
Histogram of in-degree, out-degree, and total-degree distributions for selected years. For the selected years 1995, 2003, and 2011, the first row has the in-degree distributions while the second row and the third row have the out-degree and total-degree distributions respectively. The WION is characterized by the highly left-skewed degree distributions. Most nodes enjoy high-degree connections in the WION due to the aggregated industry classification.

As shown in Fig 6, unlike other network systems such as the internet, where the degree distributions follow the power law, the WION is characterized by the highly left-skewed degree distributions. Most nodes enjoy high-degree connections in the WION because the industries are highly aggregated. Furthermore, the WION is almost complete, i.e., every node is connected with almost every node, if represented by unweighted edges. The same feature is also found in the input-output networks at the national level [12]. Using a single-year (2006) GMRIO table, Carvalho [14] also reports the heavy-tailed but non-power-law degree distributions.

**Fig 7:**
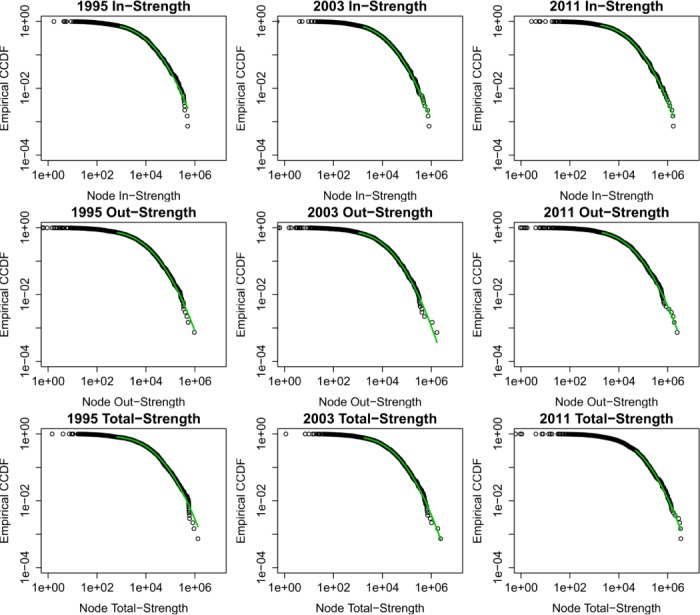
Empirical counter-cumulative distribution functions of in-strength, out-strength, and total-strength for selected years. For the selected years 1995, 2003, and 2011, the first row has the in-strength distributions while the second row and the third row have the out-strength and total-strength distributions respectively. The observed data are in black circles while the green curve is the fitted log-normal distribution.

We further take into account the edge weights and examine the strength distributions of the WION. Fig 7 shows the in-strength, out-strength, and total-strength distributions for the years 1995, 2003, and 2011. We perform Gabaix-Ibragimov test [35, 36] to see if the tails of the distributions are Pareto but find no significant power-law tails. Moreover, like the previous studies at the national level [12], the strength distributions can be well approximated by the log-normal distributions.

Our finding that the industries are both highly connected, as captured by the degree distribution, and asymmetrically connected, as captured by the strength distribution, implies that the local idiosyncratic shocks are possible to propagate through the world economy and generate a sizable global disturbance, as Acemoglu, Carvalho, Ozdaglar and Tahbaz-Salehi [37] argue that the asymmetric and fat-tailed distribution of the input-output network connections serves as the micro origin of the macro economic fluctuations. The fact that the industrial connections are increasingly globalized also calls for greater coordination and cooperation among national policy makers.

### Regional Analysis of the WION

We next explore the regional network properties of the WION by conducting community detection. A network may be partitioned into different communities, with many edges connecting nodes in the same community and few connecting nodes between different ones [38]. If the WION is completely globalized, i.e., any pair of industries in the world has an equal chance to be connected, we should not expect any significant community structure to be detected. In the following we use the modularity optimization method introduced by Newman and Girvan [39]. It is based on the idea that a random graph is not expected to have a community structure. Therefore, the possible existence of communities is revealed by the comparison between the actual density of edges in a subgraph and the expected density if the nodes are attached randomly. The expected edge density depends on the chosen null model, i.e., a copy of the original graph keeping some of its structural properties but without community structure [38].

The most popular null model, introduced by Newman and Girvan [39], keeps the degree sequence and consists of a randomized version of the original graph, where edges are rewired at random, under the constraint that the expected degree of each node matches the degree of the node in the original graph.

The modularity function to be optimized is, then, defined as:

$$\begin{array}{c} Q=\frac{1}{2m}\sum_{ij}\left(Z_{ij}-P_{ij}\right)\delta \left(C_{i},C_{j}\right) \end{array}$$ (1)

where the summation operator runs over all the node pairs. *Z* is the adjacency matrix, and *m* is the total number of edges. The *δ* function equals 1 if the two nodes *i* and *j* are in the same community and 0 otherwise. Finally, $P_{ij}=\frac{k_{i}k_{j}}{2m}$ is the probability of the presence of an edge between the two nodes *i* and *j* in the randomized null model.

**Fig 8:**
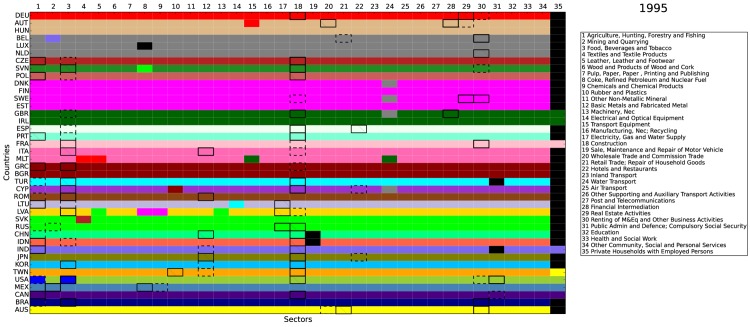
Community detection and community core detection results in 1995. The economies are arranged by rows and the industries are arranged by columns. Each color represents a community detected, except that the black color indicates the isolated nodes with only self-loop. Within each community, the top 3 core economy-industry pairs are identified. The first place is with thick and solid border. The second place is with thick and dashed border. The third place is with border and texture.

**Fig 9:**
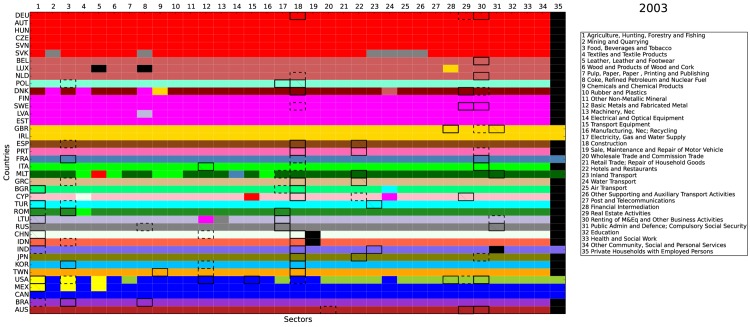
Community detection and community core detection results in 2003. The economies are arranged by rows and the industries are arranged by columns. Each color represents a community detected, except that the black color indicates the isolated nodes with only self-loop. Within each community, the top 3 core economy-industry pairs are identified. The first place is with thick and solid border. The second place is with thick and dashed border. The third place is with border and texture.

**Fig 10:**
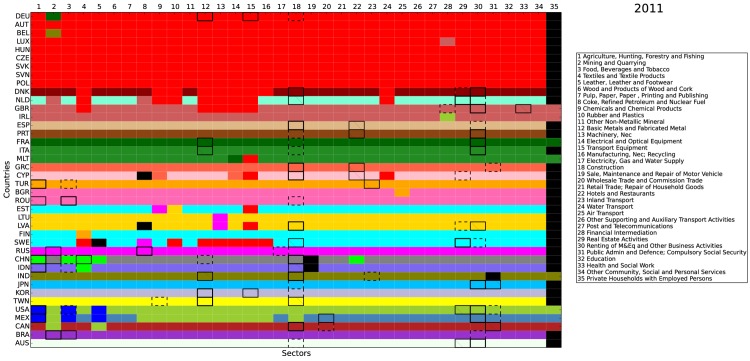
Community detection and community core detection results in 2011. The economies are arranged by rows and the industries are arranged by columns. Each color represents a community detected, except that the black color indicates the isolated nodes with only self-loop. Within each community, the top 3 core economy-industry pairs are identified. The first place is with thick and solid border. The second place is with thick and dashed border. The third place is with border and texture.

Figs 8, 9, and 10 report the community detection results for the selected years 1995, 2003, and 2011, respectively (We perform the community detection for all available years (1995–2011). Results are available upon request.). The 40 countries in the WIOD are arranged by rows while the 35 industries are arranged by columns. Different colors indicate different communities detected. There are two interesting findings in our results. First, most communities were based on a single economy, i.e., the same color often goes through a single row. This echoes one of the features of the WION mentioned in Section 1, i.e., most of the inter-industrial activities are still restricted within the country border. Second, for all the three years selected, we always color the community involving Germany in red and put it on the top. As a result, our algorithm captures a growing Germany-centered input-output community. It is centered on Germany because the community core detection results below show that the cores of this red community are all within Germany. Since the WIOD monetary goods flows are based on undeflated current prices, one possible reason for the emergence of the German community is that the community members may have experienced significantly more inflation and/or exchange rate volatility than other regions in the world. Referring to the World Bank inflation data and the exchange rate data used in the WIOD, we show that this is hardly the case (see S1 Fig).

From the above figures, it is straightforward to see that the WION is far from completely globalized. Instead, the world production is still operated nationally or at most regionally as the communities detected are either individual economies or geographically well defined regions. The detection of the emergence of the European community led by Germany is most likely the result of the production activities outsourced in recent years from Germany to Central and Eastern European countries with lower labor costs and growing demand. Another integrated community detected is in North America, which is undoubtedly fostered by the regional trade agreement, NAFTA (North American Free Trade Agreement). However, since many Asian economies are not included in the WIOD, we cannot argue if a similar trend is ongoing in the Far East. Our finding that the integration of production chains is still by and large regional implies that the governance of the global production system can be practically implemented at regional level.

Within each community, we also carry out the community core detection (see below for more technical details). In Figs 8, 9, and 10, we identify the top 3 core economy-industry pairs for each community. The first place is with thick and solid border. The second place is with thick and dashed border. The third place is with border and texture. In general, the cores are mostly concentrated in the industries of agriculture (1), mining (2), food (3), metals (12), construction (18), and financial, business, and public services (28–31). Over time, while the services industries (28–31) have become the cores in more and more developed economies, the primary industries (1–3) have become less central in the developed economies and have only remained as the cores in a few emerging economies, which is consistent with the Kuznets facts [40, 41]. Furthermore, for the growing community centered on Germany, the cores are always identified in Germany (that is why we simply call it the German community) for the three selected years. It is also worth noting that, the German industry of transport equipment (15) is identified as a core in 2011 and the car industry is the most integrated in the German community, which spans over 17 economies.

**Fig 11:**
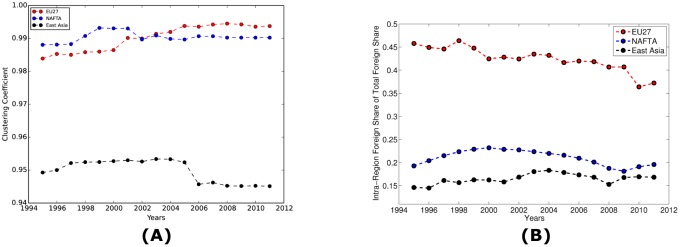
Regional clustering coefficient and intra-region foreign share of the foreign intermediate transactions over time. Panel (A) shows the average weighted clustering coefficient over time for three regions, EU27 (i.e., “Euro-Zone” and “Non-Euro EU” in S1 Table), NAFTA (see S1 Table), and East Asia (see S1 Table). EU27 and NAFTA have higher coefficients than East Asia and EU27 has an increasing trend over time, which coincides with the detection of the growing European community led by Germany. Panel (B) considers the intra-region foreign share out of the total foreign intermediate transactions for the same three regions. EU27 relies on the intra-region foreign trade the most and East Asia the least. Moreover, while the intra-region share in the other two regions is roughly stable, it has a clear decreasing trend in EU27.

Some global-level indicators can also shed light on the regional dynamics of the WION. For instance, Panel (A) in Fig 11 shows the average weighted clustering coefficient over time for three regions, EU27 (i.e., “Euro-Zone” and “Non-Euro EU” in S1 Table), NAFTA (see S1 Table), and East Asia (see S1 Table). EU27 and NAFTA have higher coefficients than East Asia and EU27 has an increasing trend over time, which coincides with the detection of the growing European community led by Germany. On the other hand, Panel (B) in Fig 11 considers the intra-region foreign share out of the total foreign intermediate transactions for the same three regions. EU27 relies on the intra-region foreign trade the most and East Asia the least. Moreover, while the intra-region share in the other two regions is roughly stable, it has a clear decreasing trend in EU27, which at first glance seems to be at odds with the detection of the growing European community led by Germany. One explanation for the contradiction is that, although the intra-region share in an absolute sense has been decreasing in EU27, relative to other regions, EU27 has been restructured in such a way that it has developed much stronger internal relationships than the external ones. Like what Zhu, Cerina, Chessa, Caldarelli, and Riccaboni [30] find in the international trade network, the peripheral countries have strong incentive to connect with Germany since it has the most external connections outside Europe. As a result, centering on Germany, the European community structure has become more hierarchical and significant over time even if the overall intra-region share of foreign intermediate transactions has been declining. Therefore, some aspects of the regional dynamics can be captured by using the global-level indicators along with the predetermined regions. However, the community detection techniques are indispensable for understanding the regional dynamics since the predetermined regions are often unavailable in practice and the global-level indicators may be oversimplified and misleading.

### Local Analysis of the WION

Finally, we conduct a local analysis of the nodes and identify the key industries in the WION by comparing the network-based measures, i.e., PageRank centrality [42] and community coreness measure [43], with the traditional methods of backward linkages, i.e., the Laumas method of backward linkages [44] and the eigenvector method of backward linkages [45]. If we use **i** to denote a summation vector of conformable size, i.e., a vector of all 1’s with the length conformable to the multiplying matrix, and let **Fi** = **f**, we then have **Zi**+**f** = **x**. Furthermore, if dividing each column of **Z** by its corresponding total output in **x**, we get the so-called technical coefficients matrix **A** (The ratios are called technical coefficients because they represent the technologies employed by the industries to transform inputs into outputs.). Replacing **Zi** with **Ax**, we rewrite the above equation as **Ax**+**f** = **x**. It can be rearranged as (**I** − **A**)**x** = **f**. Finally, we can solve **x** as follows:

$$\begin{array}{c} x={\left(I-A\right)}^{-1}f \end{array}$$ (2)

where matrix (**I** − **A**)^−1^ is often denoted by **L** and is called the Leontief inverse.

The intuition behind the Leontief inverse is that an increase in the final demand of an industry’s output will induce not only more production from the industry itself but also more from other related industries because more inputs are required. Therefore, the Leontief inverse takes into account both the direct and indirect effects of a demand increase. For instance, *L*_*ij*_ measures the total output produced in Industry *i* given a one-unit increase in Industry *j*’s final demand (The Leontief inverse is demand-driven, i.e., a repercussion effect triggered by an increase in final demand. Another strand of the input-output economics literature is based on the supply-driven model, where a repercussion effect is triggered by an increase in value added (primary inputs) [46, 47].). As a result, **i**′**L** sums up each column of **L** and each sum measures the total output of all the industries given a one-unit increase in the corresponding industry’s final demand. The vector **i**′**L** is called the Rasmussen method of backward linkages [48] and can be used to rank the industries and identify the key ones in the economy. However, as pointed out by Laumas [44], the key assumption embedded in the Rasmussen method of backward linkages is that every industry is assigned with the same weight (or unweighted), which is far from the reality. The problem with the Rasmussen method can be demonstrated by using the hypothetical data from Table 1. The calculated **i**′**L** is [2.0688 1.8377 1.2223 1.1854], which considers the industries in Economy 1 more important than the ones in Economy 2, despite the fact that Economy 2 is much larger than Economy 1 in terms of total outputs.

The industries of the 40 economies covered in the WIOD are very heterogeneous in terms of both total outputs and technical structure, which certainly makes the Rasmussen method not a good choice to identify the most central industries on a global scale. In order to identify the key industries in the WIOD, we hence use the final-demand-weighted version of the Rasmussen method, i.e., the Laumas method of backward linkages [44], which is denoted by **w** and is defined here as the Hadamard (element-wise) product of the vector of the Rasmussen backward linkages and the vector of the percentage shares of the total final demand across industries, i.e.,

$$\begin{array}{c} w=i^{\prime }L{}^{\circ}\frac{f^{\prime }}{i^{\prime }f} \end{array}$$ (3)

where ° is the element-wise multiplication operator.

Table 2 shows the top 20 industries for the years 1995, 2003, and 2011, respectively. The first column of each year is produced by the Laumas method of backward linkages, i.e., **w**. For the selected years, only four large economies, China, Germany, Japan, and USA, ever qualified for the top 20. Another noticeable change over time is the rise of China, which topped the list in 2011 with its industry of construction.

**Table 2:** Top 20 industries identified by the four methods for selected years. The first is the Laumas method of backward linkages, **w**. The second is the eigenvector method of backward linkages, **e**. The third is PageRank centrality, *PR*. The fourth is community coreness measure ∣d*Q*∣.

	1995				2003				2011			
Rank	w	e	*PR*	∣d*Q*∣	w	e	*PR*	∣d*Q*∣	w	e	*PR*	∣d*Q*∣
**1**	USA-Pub	CHN-Tpt	USA-Pub	USA-Pub	USA-Pub	CHN-Tpt	USA-Hth	USA-Obs	CHN-Cst	CHN-Tpt	GBR-Hth	CHN-Cst
**2**	JPN-Cst	CHN-Met	USA-Tpt	JPN-Cst	USA-Hth	CHN-Mch	DEU-Tpt	USA-Est	USA-Pub	CHN-Tex	DEU-Tpt	USA-Obs
**3**	USA-Cst	CHN-Mch	DEU-Tpt	USA-Obs	USA-Cst	CHN-Lth	USA-Pub	USA-Fin	USA-Hth	CHN-Elc	USA-Pub	CHN-Met
**4**	USA-Hth	CHN-Cst	USA-Hth	USA-Cst	USA-Est	CHN-Met	USA-Tpt	USA-Pub	USA-Est	CHN-Rub	CHN-Elc	USA-Pub
**5**	USA-Est	CHN-Elc	DEU-Cst	USA-Est	USA-Rtl	CHN-Tex	GBR-Hth	USA-Hth	CHN-Elc	CHN-Lth	USA-Hth	USA-Est
**6**	USA-Rtl	CHN-Rub	RUS-Hth	USA-Hth	CHN-Cst	CHN-Cst	ESP-Cst	CHN-Cst	USA-Rtl	CHN-Mch	CHN-Cst	CHN-Agr
**7**	USA-Fod	CHN-Omn	DEU-Fod	JPN-Htl	JPN-Cst	CHN-Rub	DEU-Hth	JPN-Cst	USA-Cst	CHN-Cst	CHN-Met	CHN-Fod
**8**	JPN-Pub	CHN-Lth	GBR-Cst	JPN-Met	USA-Fin	CHN-Elc	GBR-Cst	USA-Ocm	USA-Fin	CHN-Pup	USA-Tpt	USA-Fin
**9**	USA-Tpt	CHN-Mnf	USA-Cst	USA-Met	USA-Tpt	CHN-Wod	USA-Cst	CHN-Met	CHN-Fod	CHN-Wod	ESP-Cst	CHN-Min
**10**	JPN-Est	CHN-Chm	FRA-Tpt	JPN-Obs	USA-Fod	CHN-Chm	USA-Obs	USA-Met	CHN-Mch	CHN-Met	AUS-Cst	USA-Cok
**11**	JPN-Hth	CHN-Pup	USA-Fod	DEU-Cst	USA-Htl	CHN-Omn	FRA-Tpt	JPN-Obs	JPN-Cst	CHN-Chm	ITA-Hth	CHN-Elc
**12**	USA-Fin	CHN-Tex	GBR-Hth	JPN-Pub	USA-Ocm	CHN-Pup	TUR-Tex	JPN-Htl	USA-Fod	CHN-Omn	DEU-Hth	USA-Hth
**13**	USA-Htl	CHN-Hth	USA-Obs	JPN-Hth	JPN-Pub	CHN-Ait	USA-Est	CHN-Agr	USA-Htl	CHN-Ele	USA-Obs	CHN-Omn
**14**	JPN-Fod	CHN-Ait	JPN-Cst	JPN-Ocm	USA-Obs	CHN-Mnf	AUS-Cst	USA-Cst	CHN-Tpt	CHN-Ait	RUS-Hth	CHN-Cok
**15**	JPN-Rtl	CHN-Wod	DEU-Mch	JPN-Fod	JPN-Est	CHN-Wtt	USA-Fod	JPN-Pub	JPN-Pub	CHN-Mnf	CHN-Tpt	CHN-Mch
**16**	DEU-Cst	CHN-Cok	ESP-Cst	JPN-Fin	JPN-Hth	CHN-Obs	ITA-Hth	USA-Agr	USA-Tpt	CHN-Hth	DEU-Mch	USA-Cst
**17**	JPN-Elc	CHN-Wtt	JPN-Tpt	USA-Agr	USA-Whl	CHN-Hth	DEU-Cst	USA-Tpt	USA-Ocm	CHN-Obs	FRA-Cst	CHN-Chm
**18**	JPN-Whl	CHN-Ele	DEU-Met	USA-Fod	JPN-Tpt	CHN-Cok	DEU-Fod	JPN-Met	CHN-Pub	CZE-Elc	CHN-Tex	JPN-Obs
**19**	JPN-Tpt	CHN-Min	USA-Elc	JPN-Whl	CHN-Elc	CHN-Ocm	DEU-Mch	JPN-Hth	USA-Obs	CHN-Ocm	GBR-Cst	USA-Ocm
**20**	JPN-Mch	CHN-Ocm	USA-Est	USA-Pup	DEU-Tpt	CHN-Fod	CHN-Elc	CHN-Elc	USA-Whl	CHN-Min	DEU-Met	JPN-Cst

Although the Laumas method weights the industries according to their final demand, it treats their neighbors (other directly connected industries) indifferently by merely counting the total strength of linkages. This issue is addressed by the eigenvector method of backward linkages [45], which considers that an industry should have more linkages if it is connected with other industries which have more linkages. In other words, not all neighbors of an industry are equal but the one with more linkages should be weighted more. Dietzenbacher [45] captures this idea by using the left-hand eigenvector corresponding to the dominant eigenvalue of the technical coefficients matrix **A** (or equivalently the Leontief inverse **L**) as an index to rank the industries, which we denote as **e**. The ranking in the second column of each year in Table 2 is produced by the eigenvector method of backward linkages. It turns out that the top 20 industries over time are almost exclusively from China. The dominance of a single country is not surprising because, as we find in the regional analysis of the WION, industries tend to cluster on a national basis. If some industries in China, say, transportation equipment, have high linkages, almost all other industries in China will automatically receive high linkages due to the strong domestic connections and this process will reinforce itself. In this regard, the eigenvector method is problematic because the importance of some peripheral industries is clearly overestimated if they have only an insignificant connection with the real hub. Another problem of the eigenvector method is that it does not penalize the distant connections. In other words, the influence of linkages is perfectly transferred through the connections while in reality more distant connections are more likely to incur transmission loss.

The problems of the eigenvector method can be addressed by the network-based method, PageRank centrality [42]. Like the eigenvector method, PageRank centrality considers that an industry is important if it is connected with other important industries. However, PageRank centrality also contains a damping factor that penalizes the distant connections and considers that an industry is important if it is connected with other important industries and simultaneously those important industries in turn do not have other significant connections.

Since the WION is weighted, we use a weighted version of PageRank, which is computed iteratively as follows:

1. At *t* = 0, an initial probability distribution is assumed, usually $PR(i;0)=\frac{1}{N}$ where *N* is the total number of nodes;
2. At each time step, the PageRank of node *i* is computed as:
  $$\begin{array}{c} PR\left(i;t+1\right)=\frac{1-d}{N}+d\sum_{j\in M(i)}\frac{PR\left(j;t\right)w_{ij}}{S(j)} \end{array}$$ (4)
  where *M*(*i*) are the in-neighbors of *i*, *w*_*ij*_ is the weight of the link between the nodes *i* and *j*, *S* is the sum of the weights of the outgoing edges from *j*, and the damping factor *d* is set to its default value, 0.85.

In Table 2, the third column of each year is produced by PageRank centrality, which is denoted by *PR*. (Our PageRank result differs from the one reported by Carvalho [14], where he uses an unweighted version of PageRank.) Unlike the traditional methods of backward linkages, where only a few economies are among the top 20, the PageRank centrality recognizes 10 economies in the top 20 list for the three selected years.

As significant communities are detected in the regional analysis of the WION, we also consider another network-based method of identifying the key industries, i.e., community coreness measure. The idea of community coreness measure is that the nodes of a community do not have the same importance for the community stability, i.e., the removal of a node in the core of the community affects the partition much more than the deletion of a node that stays on the periphery of the community [43]. Therefore, in the following we define a novel way of detecting cores inside communities by using the properties of the modularity function, i.e., Eq (1).

By definition, if the modularity associated with a network has been optimized, every perturbation in the partition leads to a negative variation in the modularity, d*Q*. If we relocate a node from its community, we have *M* − 1 possible choices, with *M* as the number of communities, as the node’s new host community. It is possible to define the ∣d*Q*∣ associated with each node as the smallest variation in absolute value (or the closest to 0 since d*Q* is always a negative number) of all the possible choices. We call ∣d*Q*∣ the community coreness measure.

In the WION, once we have the ∣d*Q*∣ for each industry, we can consider the one with the biggest ∣d*Q*∣ the most important. We can also normalize the ∣d*Q*∣ to identify the most important nodes within each community. The results are already discussed above and are shown in Figs 8, 9, and 10, where the first place in each community is with thick and solid border, the second place is with thick dashed border, and the third place is with both border and texture.

In Table 2, the fourth column of each year is produced by community coreness measure, which is denoted again by ∣d*Q*∣. Like the Laumas method of backward linkages, the community coreness measure also includes only China, Germany, Japan, and USA in the top 20 list for the selected years.

Now we have totally four methods to identify the key industries in the WION, the Laumas method of backward linkages, the eigenvector method of backward linkages, PageRank centrality, and community coreness measure. They have different results from each other. For instance, the industry of transport equipment in Germany is captured by PageRank but not by the other three while the industry of other business activities in USA is more important by ∣d*Q*∣ than by the other three (see Table 2 and the tables of the most important economies by industry and the most important industries by economy identified by each method in Supporting Information). Table 3 reports the correlation coefficient matrix among the four methods for the selected years 1995, 2003, and 2011. We find that all the four methods are positively correlated, while **w** and ∣d*Q*∣ are correlated even more and the correlations between **e** and others are the weakest. Therefore, based on the advantages discussed above, the network-based ∣d*Q*∣ and *PR* can be used to complement, if not to substitute, **w** and **e** to identify the key industries in the WION.

**Table 3:** Correlation coefficient matrix among the four key-industry-identification methods for selected years. The first is the Laumas method of backward linkages, **w**. The second is the eigenvector method of backward linkages, **e**. The third is PageRank centrality, *PR*. The fourth is community coreness measure ∣d*Q*∣. ** and * mean that the coefficient is significant at 1% level and at 5% level respectively.

1995 (# Obs. 1400)					2003 (# Obs. 1400)					2011 (# Obs. 1400)				
	w	e	*PR*	∣d*Q*∣		w	e	*PR*	∣d*Q*∣		w	e	*PR*	∣d*Q*∣
**w**	1	-	-	-	**w**	1	-	-	-	**w**	1	-	-	-
**e**	0.030	1	-	-	**e**	0.060*	1	-	-	**e**	0.258**	1	-	-
*PR*	0.664**	0.042	1	-	*PR*	0.689**	0.102**	1	-	*PR*	0.643**	0.249**	1	-
∣d*Q*∣	0.820**	0.127**	0.650**	1	∣d*Q*∣	0.724**	0.207**	0.596**	1	∣d*Q*∣	0.754**	0.337**	0.592**	1

## Discussion

This paper investigates a GMRIO system characterized by the recently available WIOD database. By viewing the world input-output system as an interdependent network where the nodes are the individual industries in different economies and the edges are the monetary goods flows between industries, we study the network properties of the so-called world input-output network (WION) at global, regional, and local levels, and document its evolution over time.

We first quantify some global network properties of the WION such as assortativity, clustering coefficient, and degree and strength distributions. We find that both its assortativity and clustering coefficient are positively correlated with the foreign share of the intermediate transactions over time. In other words, the increasingly integrated production chains tend to make the WION more clustered and more assortative. We also observe that the 2008–2009 financial crisis creates a significant and disruptive effect on the global production chains and makes the WION less clustered and less assortative. Furthermore, we find that the degree distribution of the WION is highly left-skewed, which is the consequence of the highly aggregated industry classification. On the other hand, the strength distribution of the WION is fat-tailed (log-normal). The fact that the industries are both highly connected, as captured by the degree distribution, and asymmetrically connected, as captured by the strength distribution, implies that the local idiosyncratic shocks are possible to propagate through the world economy and generate a sizable global fluctuation, as vividly illustrated by the recent financial crisis. The increasing foreign share of the intermediate transactions also implies the interdependence among national policies. That is, policy makers have to be aware of the possibility that their industry-specific policies can have not only an impact on the intended industry, but also unintended consequences on seemingly unrelated domestic and foreign markets, from which the intended industry typically sources goods and services.

We then focus on its subgraph structure and dynamics by using community detection techniques. Even though most of the time the foreign share of the intermediate transactions has been increasing, the integration of production chains is by and large regional rather than global. That is, the world production is still operated nationally or at most regionally as the communities detected are either individual economies or geographically well defined regions. In particular, we detect the emergence of a large European community led by Germany, which is most likely the result of the production activities outsourced in recent years from Germany to Central and Eastern European countries with lower labor costs and growing demand. This finding also implies that the governance of the global production system can be practically implemented at regional level and its success depends on a good understanding of the asymmetrical roles played by different industries in different economies within the same region.

Finally, we quantify some local network properties of the WION and compare them with the traditional methods of input-output analysis to identify the key industries. The traditional methods we consider are the Laumas method of backward linkages [44] and the eigenvector method of backward linkages [45] while the network-based methods we consider are PageRank centrality [42] and community coreness measure [43]. We find that the methods rank the industries differently because they capture different aspects of the connections. Nevertheless, we argue that the network-based measures can give valuable insights into identifying the key industries. At first glance, it may seem that the eigenvector method of backward linkages and PageRank centrality share the same spirit since both of them consider that an industry is important if it is connected with other important industries. However, two potential problems with the eigenvector method of backward linkages are that if an important industry is connected with many industries then all those industries will automatically become “important” while in reality they may be just peripheral industries and that it does not penalize the distant connections while in reality the distant connections are often associated with transmission loss. Unlike the eigenvector method of backward linkages, PageRank centrality contains a damping factor that penalizes the distant connections and considers that an industry is important if it is connected with other important industries and simultaneously those important industries in turn do not have other significant connections. On the other hand, community coreness measure considers that an industry is more important if it matters more in terms of reserving the community structure of the network. Therefore, special attention should be given to the industries with high community coreness when policy makers seek to understand and to restructure the production chains.

We conclude the paper by discussing some future extensions. As mentioned above, due to the limited coverage of the WIOD, we cannot argue if the input-output integration is also observed in other continents. Therefore, in our future work, we plan to use some GMRIO databases with better coverage. One possible choice is the EORA [49, 50], which covers about 187 countries in the world and the years from 1990 to 2011. Moreover, although we have shown that the network approach can be used as a complement to the conventional input-output methods to retrieve more information from the input-output tables, a more systematic comparison between input-output analysis and network analysis is needed to foster fruitful interaction between the two.

## Supporting Information

S1 Fig Average inflation rate and exchange rate. Panel (a) shows the average inflation rate of all the 40 WIOD economies (except Taiwan and Bulgaria) versus the average inflation rate of the German community. We compare the average inflation rate, i.e., the annual GDP deflator, across all the WIOD economies (except Taiwan and Bulgaria) with the average annual GDP deflator across the 9 major member economies in the German community detected in 2011, i.e., Germany, Austria, Belgium, Luxembourg, Hungary, Czech Republic, Slovakia, Slovenia, and Poland. During 1995–2011, the average inflation of the German community was almost always below that of all the WIOD economies. The data source is the World Development Indicators, the World Bank. Panel (b) shows the average exchange rate of all the 40 WIOD economies versus the average exchange rate of the German community. We compare the average exchange rate, i.e., US dollars per unit of local currency, across all the WIOD economies with the average exchange rate across the 9 major economies in the German community detected in 2011, i.e., Germany, Austria, Belgium, Luxembourg, Hungary, Czech Republic, Slovakia, Slovenia, and Poland. The average exchange rate of the German community was basically below that of all the WIOD economies before 2000. Only from 2001, the community average became slightly (no more than 16%) higher than the overall average. The data source is the exchange rate data used in the WIOD. Therefore, the emergence of the German community cannot be attributed to inflation or exchange rate dynamics.
(PDF)

S1 Table List of WIOD economies. (PDF)

S2 Table List of WIOD industries. (PDF)

S3 Table The most important economies by industry over time: using the Laumas method of backward linkages. The codes of countries and industries can be found in S1 Table and S2 Table.
(PDF)

S4 Table The most important industries by economy over time: using the Laumas method of backward linkages. The codes of countries and industries can be found in S1 Table and S2 Table.
(PDF)

S5 Table The most important economies by industry over time: using the eigenvector method of backward linkages. The codes of countries and industries can be found in S1 Table and S2 Table.
(PDF)

S6 Table The most important industries by economy over time: using the eigenvector method of backward linkages. The codes of countries and industries can be found in S1 Table and S2 Table.
(PDF)

S7 Table The most important economies by industry over time: using PageRank centrality. The codes of countries and industries can be found in S1 Table and S2 Table.
(PDF)

S8 Table The most important industries by economy over time: using PageRank centrality. The codes of countries and industries can be found in S1 Table and S2 Table.
(PDF)

S9 Table The most important economies by industry over time: using community coreness measure. The codes of countries and industries can be found in S1 Table and S2 Table.
(PDF)

S10 Table The most important industries by economy over time: using community coreness measure. The codes of countries and industries can be found in S1 Table and S2 Table.
(PDF)
