# Supplementary figures and images for: World Input-Output Network
Source: PLoS One. 2015 Jul 29;10(7):e0134025. doi: 10.1371/journal.pone.0134025 (PMC4519177; doi:10.1371/journal.pone.0134025)

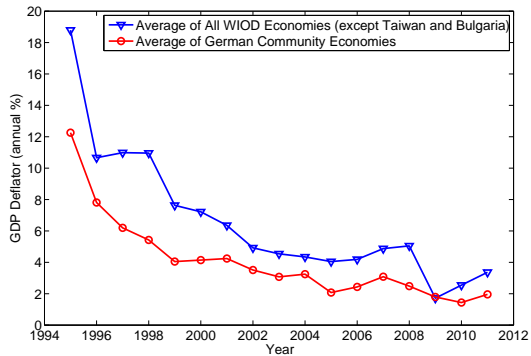

(a) Inflation

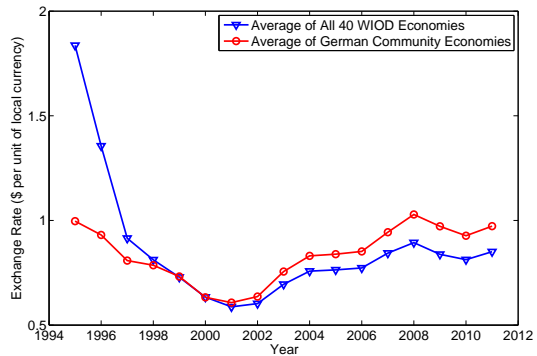

(b) Exchange Rate

Supplement: S1 Fig — Panel (a) shows the average inflation rate of all the 40 WIOD economies (except Taiwan and Bulgaria) versus the average inflation rate of the German community. We compare the average inflation rate, i.e., the annual GDP deflator, across all the WIOD economies (except Taiwan and Bulgaria) with the average annual GDP deflator across the 9 major member economies in the German community detected in 2011, i.e., Germany, Austria, Belgium, Luxembourg, Hungary, Czech Republic, Slovakia, Slovenia, and Poland. During 1995–2011, the average inflation of the German community was almost always below that of all the WIOD economies. The data source is the World Development Indicators, the World Bank. Panel (b) shows the average exchange rate of all the 40 WIOD economies versus the average exchange rate of the German community. We compare the average exchange rate, i.e., US dollars per unit of local currency, across all the WIOD economies with the average exchange rate across the 9 major economies in the German community detected in 2011, i.e., Germany, Austria, Belgium, Luxembourg, Hungary, Czech Republic, Slovakia, Slovenia, and Poland. The average exchange rate of the German community was basically below that of all the WIOD economies before 2000. Only from 2001, the community average became slightly (no more than 16%) higher than the overall average. The data source is the exchange rate data used in the WIOD. Therefore, the emergence of the German community cannot be attributed to inflation or exchange rate dynamics. (PDF) [file pone.0134025.s001.pdf]
