# Supplementary material for: World Input-Output Network
Source: PLoS One. 2015 Jul 29;10(7):e0134025. doi: 10.1371/journal.pone.0134025 (PMC4519177; doi:10.1371/journal.pone.0134025)
Supplement: S4 Table — The codes of countries and industries can be found in S1 Table and S2 Table. (PDF) [file pone.0134025.s005.pdf]

Economy/Year	1995	1996	1997	1998	1999	2000	2001	2002	2003	2004	2005	2006	2007	2008	2009	2010	2011
**AUS**	Cst	Cst	Cst	Cst	Cst	Cst	Cst	Cst	Cst	Cst	Cst	Cst	Cst	Cst	Cst	Cst	Cst
**AUT**	Cst	Cst	Cst	Cst	Cst	Cst	Cst	Cst	Cst	Cst	Cst	Cst	Cst	Cst	Cst	Cst	Cst
**BEL**	Fod	Fod	Cst	Cst	Cst	Cst	Cst	Cst	Cst	Cst	Cst	Cst	Cst	Cst	Cst	Cst	Cst
**BGR**	Fod	Fod	Fod	Fod	Agr	Agr	Fod	Fod	Fod	Fod	Cst	Cst	Cst	Cst	Cst	Cst	Cst
**BRA**	Pub	Pub	Pub	Pub	Pub	Pub	Pub	Pub	Pub	Pub	Pub	Pub	Pub	Pub	Pub	Pub	Pub
**CAN**	Pub	Pub	Cst	Pub	Tpt	Tpt	Pub	Pub	Cst	Cst	Cst	Cst	Cst	Cst	Cst	Cst	Cst
**CHN**	Cst	Cst	Cst	Cst	Cst	Cst	Cst	Cst	Cst	Cst	Cst	Cst	Cst	Cst	Cst	Cst	Cst
**CYP**	Htl	Cst	Htl	Htl	Htl	Htl	Htl	Pub	Pub	Cst	Cst	Cst	Cst	Cst	Cst	Cst	Cst
**CZE**	Cst	Cst	Cst	Cst	Cst	Cst	Cst	Cst	Cst	Cst	Cst	Cst	Cst	Cst	Cst	Tpt	Tpt
**DEU**	Cst	Cst	Cst	Cst	Cst	Cst	Tpt	Tpt	Tpt	Tpt	Tpt	Tpt	Tpt	Tpt	Tpt	Tpt	Tpt
**DNK**	Fod	Fod	Cst	Cst	Cst	Cst	Cst	Hth	Hth	Hth	Cst	Cst	Cst	Hth	Hth	Hth	Hth
**ESP**	Cst	Cst	Cst	Cst	Cst	Cst	Cst	Cst	Cst	Cst	Cst	Cst	Cst	Cst	Cst	Cst	Cst
**EST**	Fod	Fod	Fod	Cst	Cst	Cst	Cst	Cst	Cst	Cst	Cst	Cst	Cst	Cst	Cst	Cst	Cst
**FIN**	Cst	Cst	Cst	Cst	Cst	Cst	Cst	Cst	Cst	Cst	Cst	Cst	Cst	Cst	Cst	Cst	Cst
**FRA**	Cst	Cst	Cst	Cst	Cst	Cst	Cst	Cst	Cst	Cst	Cst	Cst	Cst	Cst	Cst	Cst	Cst
**GBR**	Pub	Cst	Cst	Cst	Cst	Cst	Cst	Cst	Cst	Cst	Hth	Hth	Hth	Hth	Hth	Hth	Hth
**GRC**	Cst	Cst	Cst	Cst	Cst	Cst	Cst	Cst	Cst	Cst	Cst	Cst	Cst	Cst	Pub	Pub	Pub
**HUN**	Fod	Fod	Fod	Fod	Elc	Elc	Elc	Elc	Elc	Elc	Elc	Elc	Elc	Elc	Elc	Elc	Elc
**IDN**	Cst	Cst	Cst	Cst	Fod	Cst	Cst	Cst	Cst	Cst	Cst	Cst	Cst	Cst	Cst	Cst	Cst
**IND**	Agr	Agr	Cst	Cst	Cst	Cst	Cst	Cst	Cst	Cst	Cst	Cst	Cst	Cst	Cst	Cst	Cst
**IRL**	Fod	Fod	Fod	Elc	Elc	Elc	Elc	Elc	Cst	Cst	Cst	Cst	Cst	Cst	Cst	Chm	Chm
**ITA**	Cst	Cst	Cst	Cst	Cst	Cst	Cst	Cst	Cst	Cst	Cst	Cst	Cst	Cst	Cst	Cst	Cst
**JPN**	Cst	Cst	Cst	Cst	Cst	Cst	Cst	Cst	Cst	Cst	Cst	Cst	Cst	Cst	Cst	Cst	Cst
**KOR**	Cst	Cst	Cst	Cst	Cst	Cst	Cst	Cst	Cst	Cst	Cst	Cst	Cst	Cst	Cst	Cst	Cst
**LTU**	Fod	Fod	Pub	Cst	Cst	Fod	Fod	Cst	Cst	Cst	Cst	Cst	Cst	Cst	Fod	Fod	Fod
**LUX**	Cst	Fin	Fin	Fin	Fin	Fin	Fin	Fin	Fin	Fin	Fin	Fin	Fin	Fin	Fin	Fin	Fin
**LVA**	Fod	Fod	Fod	Cst	Cst	Cst	Cst	Cst	Cst	Cst	Cst	Cst	Cst	Cst	Cst	Cst	Cst
**MEX**	Fod	Fod	Fod	Fod	Fod	Cst	Cst	Cst	Cst	Cst	Cst	Cst	Cst	Cst	Cst	Cst	Cst
**MLT**	Elc	Elc	Elc	Elc	Elc	Elc	Elc	Cst	Cst	Elc	Cst	Ocm	Ocm	Ocm	Ocm	Ocm	Ocm
**NLD**	Fod	Fod	Fod	Fod	Cst	Cst	Cst	Cst	Pub	Pub	Pub	Pub	Cst	Cst	Pub	Pub	Pub
**POL**	Fod	Fod	Fod	Cst	Cst	Cst	Cst	Fod	Fod	Fod	Fod	Fod	Cst	Cst	Cst	Cst	Cst
**PRT**	Cst	Cst	Cst	Cst	Cst	Cst	Cst	Cst	Cst	Cst	Cst	Cst	Cst	Cst	Cst	Cst	Cst
**ROM**	Fod	Fod	Fod	Fod	Fod	Fod	Fod	Fod	Fod	Fod	Cst	Cst	Cst	Cst	Cst	Cst	Cst
**RUS**	Fod	Cst	Cst	Cst	Fod	Fod	Cst	Cst	Cst	Cst	Cst	Cst	Cst	Cst	Cst	Cst	Cst
**SVK**	Cst	Cst	Cst	Cst	Cst	Cst	Tpt	Tpt	Tpt	Tpt	Cst	Tpt	Tpt	Tpt	Cst	Cst	Cst
**SVN**	Cst	Cst	Cst	Cst	Cst	Cst	Cst	Cst	Cst	Cst	Cst	Cst	Cst	Cst	Cst	Cst	Cst
**SWE**	Est	Est	Est	Est	Est	Hth	Hth	Hth	Hth	Hth	Hth	Hth	Hth	Hth	Hth	Hth	Hth
**TUR**	Cst	Cst	Cst	Cst	Fod	Fod	Fod	Tex	Tex	Tex	Fod	Fod	Tex	Fod	Fod	Fod	Fod
**TWN**	Cst	Cst	Pub	Pub	Elc	Elc	Elc	Elc	Elc	Cst	Cst	Cst	Cst	Cst	Cst	Cst	Cst
**USA**	Pub	Pub	Pub	Pub	Pub	Pub	Pub	Pub	Pub	Pub	Pub	Pub	Pub	Pub	Pub	Pub	Pub
