# Supplementary material for: World Input-Output Network
Source: PLoS One. 2015 Jul 29;10(7):e0134025. doi: 10.1371/journal.pone.0134025 (PMC4519177; doi:10.1371/journal.pone.0134025)
Supplement: S6 Table — The codes of countries and industries can be found in S1 Table and S2 Table. (PDF) [file pone.0134025.s007.pdf]

Economy/Year	1995	1996	1997	1998	1999	2000	2001	2002	2003	2004	2005	2006	2007	2008	2009	2010	2011
**AUS**	Tex	Tex	Tex	Tex	Tex	Tex	Wtt	Tex	Lth	Mch	Mch	Mch	Mch	Mch	Mch	Mch	Mch
**AUT**	Wtt	Wtt	Tpt	Tpt	Tpt	Tpt	Elc	Tpt	Tpt	Tpt	Tpt	Tpt	Ele	Ele	Ele	Ele	Ele
**BEL**	Tpt	Tpt	Tex	Tex	Tpt	Tex	Wtt	Tpt	Tex	Tpt	Tpt	Tpt	Tpt	Tpt	Tex	Tex	Tex
**BGR**	Elc	Elc	Elc	Elc	Elc	Elc	Elc	Cok	Elc	Elc	Elc	Elc	Elc	Elc	Elc	Elc	Elc
**BRA**	Tex	Elc	Elc	Elc	Elc	Elc	Elc	Elc	Elc	Elc	Elc	Elc	Elc	Elc	Elc	Elc	Elc
**CAN**	Tex	Tex	Cok	Tex	Tex	Tex	Elc	Elc	Elc	Tex	Tex	Tex	Tex	Tex	Tex	Tex	Tex
**CHN**	Tpt	Tpt	Tpt	Tpt	Tpt	Tpt	Tpt	Tpt	Tpt	Tpt	Tpt	Tpt	Tpt	Tpt	Tpt	Tpt	Tpt
**CYP**	Tex	Tex	Tex	Tex	Tex	Tex	Elc	Tex	Tex	Tex	Tex	Elc	Met	Cok	Elc	Elc	Elc
**CZE**	Tpt	Elc	Tpt	Elc	Elc	Tpt	Elc	Elc	Elc	Elc	Elc	Elc	Elc	Elc	Elc	Elc	Elc
**DEU**	Tex	Tex	Tex	Tex	Tpt	Tpt	Elc	Tpt	Elc	Tpt	Elc	Elc	Elc	Elc	Tex	Elc	Elc
**DNK**	Wtt	Wtt	Wtt	Wtt	Wtt	Tex	Wtt	Tex	Tex	Tpt	Tex	Tex	Tex	Tex	Ait	Tpt	Tpt
**ESP**	Tpt	Tpt	Tpt	Tpt	Tpt	Tpt	Elc	Tpt	Tpt	Tpt	Elc	Elc	Elc	Elc	Elc	Elc	Tex
**EST**	Elc	Elc	Elc	Elc	Elc	Elc	Elc	Elc	Elc	Elc	Elc	Elc	Elc	Tex	Elc	Elc	Tex
**FIN**	Elc	Elc	Elc	Elc	Elc	Elc	Elc	Elc	Tpt	Elc	Elc	Elc	Elc	Elc	Elc	Elc	Elc
**FRA**	Tpt	Tpt	Tpt	Tpt	Tpt	Tpt	Tpt	Tpt	Tpt	Tpt	Tpt	Tpt	Tpt	Tpt	Elc	Tpt	Elc
**GBR**	Elc	Elc	Elc	Elc	Tpt	Elc	Wod	Elc	Elc	Tpt	Tpt	Elc	Elc	Elc	Elc	Elc	Elc
**GRC**	Met	Tex	Tex	Tex	Tex	Tex	Elc	Met	Tpt	Met	Met	Met	Met	Met	Met	Met	Met
**HUN**	Tpt	Tpt	Elc	Elc	Elc	Elc	Elc	Elc	Elc	Elc	Elc	Elc	Elc	Elc	Elc	Elc	Elc
**IDN**	Tex	Tex	Tex	Tex	Tex	Tex	Ait	Tex	Tex	Mch	Mch	Mch	Mch	Tex	Tex	Tex	Tex
**IND**	Mnf	Mnf	Mnf	Mnf	Mnf	Mnf	Mnf	Mnf	Mnf	Mnf	Mnf	Mnf	Mnf	Mnf	Mnf	Mnf	Mnf
**IRL**	Wtt	Wtt	Wtt	Elc	Elc	Elc	Wod	Elc	Elc	Elc	Elc	Elc	Elc	Elc	Elc	Elc	Elc
**ITA**	Tex	Tex	Tex	Tex	Tpt	Tpt	Tpt	Tpt	Tpt	Tpt	Tpt	Tpt	Tpt	Tpt	Tex	Tpt	Tpt
**JPN**	Tpt	Tpt	Tpt	Tpt	Tpt	Tpt	Tpt	Tpt	Tpt	Tpt	Tpt	Tpt	Tpt	Tpt	Tpt	Tpt	Tpt
**KOR**	Tex	Tex	Met	Tex	Tex	Met	Tpt	Tpt	Met	Met	Tpt	Tpt	Tpt	Tpt	Tpt	Tpt	Tpt
**LTU**	Tex	Elc	Tex	Tex	Tex	Tex	Elc	Elc	Elc	Elc	Elc	Elc	Elc	Elc	Tex	Tex	Tex
**LUX**	Met	Met	Met	Met	Met	Met	Wtt	Met	Mch	Elc	Elc	Ocm	Tpt	Met	Ait	Tpt	Tex
**LVA**	Tex	Tex	Tex	Tex	Tex	Tex	Elc	Elc	Elc	Elc	Elc	Elc	Met	Met	Tex	Tex	Tex
**MEX**	Elc	Elc	Elc	Elc	Elc	Elc	Elc	Elc	Elc	Elc	Elc	Elc	Elc	Elc	Elc	Elc	Elc
**MLT**	Elc	Elc	Elc	Elc	Elc	Elc	Wtt	Elc	Elc	Elc	Elc	Elc	Elc	Elc	Elc	Elc	Elc
**NLD**	Tpt	Tpt	Tpt	Tex	Tex	Tex	Elc	Tex	Tex	Tex	Tex	Tex	Tex	Tex	Tex	Tex	Tex
**POL**	Elc	Tpt	Tpt	Tpt	Tpt	Tpt	Ait	Elc	Elc	Elc	Elc	Elc	Elc	Elc	Tex	Elc	Elc
**PRT**	Elc	Elc	Tpt	Elc	Tpt	Tpt	Elc	Tpt	Elc	Elc	Elc	Elc	Elc	Elc	Elc	Elc	Elc
**ROM**	Ele	Met	Chm	Chm	Chm	Chm	Ele	Ele	Ele	Met	Elc	Tex	Met	Elc	Elc	Wtt	Elc
**RUS**	Tex	Tex	Tex	Tex	Tex	Tex	Tex	Tex	Tex	Tex	Tpt	Tpt	Tpt	Tpt	Tex	Tex	Tex
**SVK**	Tpt	Ait	Tpt	Lth	Elc	Ele	Ele	Ele	Ele	Elc	Elc	Elc	Elc	Elc	Elc	Elc	Elc
**SVN**	Tpt	Tpt	Tpt	Tpt	Tpt	Tpt	Tpt	Tpt	Tex	Tpt	Tpt	Tpt	Tpt	Tex	Tex	Tex	Tex
**SWE**	Wtt	Wtt	Elc	Elc	Elc	Elc	Elc	Elc	Elc	Tpt	Tpt	Elc	Elc	Elc	Elc	Elc	Elc
**TUR**	Tex	Tex	Tex	Tex	Elc	Elc	Elc	Tex	Tex	Elc	Elc	Elc	Elc	Tex	Tex	Tex	Tex
**TWN**	Mch	Mch	Mch	Met	Mch	Mch	Elc	Elc	Mch	Mch	Mch	Mch	Elc	Elc	Elc	Elc	Elc
**USA**	Lth	Lth	Lth	Lth	Lth	Lth	Lth	Lth	Lth	Tpt	Lth	Lth	Tpt	Tpt	Tpt	Tpt	Tpt
