# Supplementary material for: World Input-Output Network
Source: PLoS One. 2015 Jul 29;10(7):e0134025. doi: 10.1371/journal.pone.0134025 (PMC4519177; doi:10.1371/journal.pone.0134025)
Supplement: S7 Table — The codes of countries and industries can be found in S1 Table and S2 Table. (PDF) [file pone.0134025.s008.pdf]

Industry/Year	1995	1996	1997	1998	1999	2000	2001	2002	2003	2004	2005	2006	2007	2008	2009	2010	2011
**Agr**	RUS	RUS	RUS	RUS	DEU	BGR	BGR	CHN	CHN	CHN	CHN	CHN	CHN	RUS	CHN	CHN	CHN
**Min**	RUS	RUS	RUS	RUS	RUS	RUS	RUS	RUS	RUS	RUS	RUS	RUS	RUS	RUS	RUS	RUS	RUS
**Fod**	DEU	DEU	DEU	USA	DEU	USA	USA	USA	USA	USA	USA	USA	USA	USA	USA	USA	USA
**Tex**	ITA	ITA	ITA	ITA	ITA	TUR	ITA	TUR	TUR	TUR	TUR	TUR	TUR	TUR	TUR	TUR	CHN
**Lth**	ITA	ITA	ITA	ITA	ITA	ITA	ITA	CHN	CHN	CHN	ITA	ITA	ITA	ITA	CHN	CHN	CHN
**Wod**	DEU	DEU	USA	USA	USA	USA	USA	USA	LVA	USA	USA	USA	LVA	CHN	CHN	CHN	CHN
**Pup**	USA	USA	USA	USA	USA	USA	USA	USA	USA	USA	USA	USA	USA	USA	USA	USA	USA
**Cok**	BRA	USA	BRA	BRA	BRA	USA	USA	USA	DEU	DEU	USA	USA	USA	RUS	RUS	USA	FRA
**Chm**	DEU	USA	USA	USA	USA	USA	USA	USA	USA	USA	USA	USA	USA	USA	USA	USA	CHN
**Rub**	DEU	DEU	USA	DEU	USA	USA	USA	USA	USA	DEU	DEU	DEU	DEU	CHN	CHN	CHN	CHN
**Omn**	CHN	CHN	CHN	CHN	CHN	CHN	CHN	CHN	CHN	CHN	CHN	CHN	CHN	CHN	CHN	CHN	CHN
**Met**	DEU	DEU	DEU	DEU	USA	DEU	DEU	DEU	DEU	DEU	DEU	CHN	CHN	CHN	CHN	CHN	CHN
**Mch**	DEU	DEU	DEU	DEU	DEU	DEU	DEU	DEU	DEU	DEU	DEU	DEU	DEU	DEU	DEU	DEU	DEU
**Elc**	USA	USA	USA	USA	USA	USA	DEU	DEU	CHN	CHN	CHN	CHN	CHN	CHN	CHN	CHN	CHN
**Tpt**	USA	DEU	USA	DEU	DEU	DEU	DEU	DEU	DEU	DEU	DEU	DEU	DEU	DEU	DEU	DEU	DEU
**Mnf**	DEU	DEU	ITA	DEU	ITA	DEU	DEU	ITA	ITA	DEU	DEU	DEU	DEU	DEU	DEU	DEU	DEU
**Ele**	FRA	FRA	RUS	FRA	DEU	USA	USA	DEU	DEU	DEU	DEU	DEU	DEU	DEU	DEU	RUS	RUS
**Cst**	DEU	DEU	DEU	USA	USA	USA	USA	ESP	ESP	ESP	ESP	ESP	ESP	ESP	ESP	CHN	CHN
**Sal**	ROM	ROM	ROM	ROM	ROM	ROM	ROM	ROM	ITA	ITA	ITA	ITA	ITA	ITA	ITA	ITA	ITA
**Whl**	ITA	ITA	ITA	ITA	ITA	ITA	ITA	ITA	ITA	ITA	ITA	ITA	RUS	RUS	RUS	RUS	RUS
**Rtl**	USA	USA	USA	USA	USA	USA	GBR	GBR	GBR	GBR	USA	USA	GBR	USA	GBR	USA	USA
**Htl**	USA	USA	USA	USA	USA	USA	USA	USA	USA	USA	USA	USA	USA	USA	USA	USA	USA
**Ldt**	IND	IND	IND	IND	IND	IND	IND	IND	IND	IND	IND	IND	IND	IND	IND	IND	IND
**Wtt**	JPN	JPN	JPN	JPN	JPN	JPN	JPN	DNK	JPN	JPN	JPN	JPN	JPN	JPN	JPN	JPN	JPN
**Ait**	CYP	CYP	CYP	CYP	CYP	CYP	CYP	CYP	CYP	CYP	CYP	DEU	DEU	DEU	DEU	DEU	DEU
**Otr**	DEU	DEU	DEU	DEU	DEU	DEU	DEU	DEU	SWE	DEU	DEU	DEU	DEU	DEU	DEU	SWE	SWE
**Pst**	USA	USA	USA	USA	USA	KOR	USA	USA	USA	USA	USA	USA	USA	USA	USA	USA	USA
**Fin**	USA	USA	USA	USA	USA	USA	USA	USA	USA	USA	USA	USA	USA	USA	USA	USA	USA
**Est**	USA	USA	USA	USA	USA	USA	USA	USA	USA	USA	USA	USA	USA	USA	USA	USA	USA
**Obs**	USA	USA	USA	USA	USA	USA	USA	USA	USA	USA	USA	USA	USA	USA	USA	USA	USA
**Pub**	USA	USA	USA	USA	USA	USA	USA	USA	USA	USA	USA	USA	USA	USA	USA	USA	USA
**Edu**	RUS	RUS	RUS	RUS	GBR	GBR	GBR	GBR	CHN	GBR	CHN	CHN	CHN	CHN	DEU	CHN	CHN
**Hth**	USA	USA	USA	USA	USA	USA	USA	USA	USA	USA	GBR	GBR	GBR	GBR	GBR	GBR	GBR
**Ocm**	USA	USA	USA	USA	USA	USA	USA	USA	USA	USA	USA	USA	USA	USA	USA	USA	USA
**Pvt**	IND	IND	IND	IND	IND	IND	IND	IND	IND	IND	IND	IND	IND	IND	IND	IND	IND
