# Supplementary material for: World Input-Output Network
Source: PLoS One. 2015 Jul 29;10(7):e0134025. doi: 10.1371/journal.pone.0134025 (PMC4519177; doi:10.1371/journal.pone.0134025)
Supplement: S8 Table — The codes of countries and industries can be found in S1 Table and S2 Table. (PDF) [file pone.0134025.s009.pdf]

Economy/Year	1995	1996	1997	1998	1999	2000	2001	2002	2003	2004	2005	2006	2007	2008	2009	2010	2011
**AUS**	Cst	Cst	Cst	Cst	Cst	Cst	Cst	Cst	Cst	Cst	Cst	Cst	Cst	Cst	Cst	Cst	Cst
**AUT**	Cst	Cst	Cst	Cst	Cst	Cst	Cst	Cst	Hth	Cst	Cst	Cst	Cst	Cst	Cst	Cst	Cst
**BEL**	Hth	Hth	Hth	Hth	Cst	Hth	Cst	Hth	Hth	Hth	Cst	Cst	Cst	Cst	Cst	Cst	Cst
**BGR**	Fod	Fod	Agr	Agr	Agr	Agr	Agr	Agr	Agr	Agr	Agr	Agr	Fod	Cst	Cst	Cst	Cst
**BRA**	Fod	Fod	Fod	Fod	Fod	Fod	Fod	Fod	Fod	Fod	Fod	Fod	Tpt	Tpt	Fod	Tpt	Tpt
**CAN**	Pub	Pub	Pub	Pub	Pub	Pub	Pub	Pub	Pub	Pub	Pub	Pub	Pub	Pub	Pub	Tpt	Tpt
**CHN**	Tex	Tex	Met	Tex	Tex	Elc	Tex	Elc	Elc	Elc	Elc	Elc	Elc	Elc	Elc	Elc	Elc
**CYP**	Fod	Fod	Fod	Pub	Fod	Fod	Pub	Fod	Fod	Fod	Pub	Pub	Pub	Cst	Pub	Pub	Pub
**CZE**	Cst	Cst	Cst	Cst	Cst	Cst	Cst	Cst	Cst	Cst	Cst	Cst	Cst	Cst	Cst	Tpt	Tpt
**DEU**	Tpt	Tpt	Tpt	Tpt	Tpt	Tpt	Tpt	Tpt	Tpt	Tpt	Tpt	Tpt	Tpt	Tpt	Tpt	Tpt	Tpt
**DNK**	Fod	Fod	Fod	Fod	Fod	Fod	Fod	Fod	Fod	Fod	Fod	Obs	Obs	Obs	Obs	Obs	Obs
**ESP**	Cst	Cst	Cst	Cst	Cst	Cst	Cst	Cst	Cst	Cst	Cst	Cst	Cst	Cst	Cst	Cst	Cst
**EST**	Fod	Fod	Fod	Fod	Otr	Otr	Otr	Otr	Otr	Otr	Otr	Cst	Cst	Cst	Otr	Otr	Otr
**FIN**	Pup	Pup	Pup	Cst	Cst	Elc	Cst	Cst	Cst	Cst	Cst	Hth	Hth	Hth	Hth	Hth	Hth
**FRA**	Tpt	Tpt	Tpt	Tpt	Tpt	Tpt	Tpt	Tpt	Tpt	Tpt	Tpt	Tpt	Tpt	Tpt	Tpt	Tpt	Cst
**GBR**	Cst	Cst	Cst	Cst	Hth	Hth	Hth	Hth	Hth	Hth	Hth	Hth	Hth	Hth	Hth	Hth	Hth
**GRC**	Pub	Fod	Fod	Fod	Pub	Pub	Pub	Fod	Cst	Fod	Fod	Cst	Ocm	Fod	Ocm	Ocm	Ocm
**HUN**	Fod	Fod	Fod	Fod	Fod	Fod	Fod	Fod	Fod	Fod	Fod	Fod	Fod	Fod	Fod	Fod	Agr
**IDN**	Cst	Cst	Tex	Fod	Fod	Fod	Fod	Fod	Fod	Cst	Cst	Cst	Cst	Cst	Cst	Cst	Cst
**IND**	Fod	Ldt	Ldt	Ldt	Ldt	Fod	Ldt	Fod	Fod	Cst	Cst	Cst	Cst	Cst	Cst	Cst	Cst
**IRL**	Cst	Cst	Cst	Cst	Cst	Cst	Cst	Cst	Cst	Cst	Cst	Cst	Cst	Cst	Hth	Hth	Hth
**ITA**	Cst	Cst	Cst	Tex	Tex	Tex	Tex	Hth	Hth	Hth	Hth	Hth	Hth	Hth	Hth	Hth	Hth
**JPN**	Cst	Cst	Tpt	Cst	Cst	Tpt	Tpt	Tpt	Tpt	Tpt	Tpt	Tpt	Tpt	Tpt	Tpt	Tpt	Tpt
**KOR**	Tpt	Tpt	Tpt	Tpt	Tpt	Tpt	Tpt	Tpt	Tpt	Tpt	Tpt	Tpt	Tpt	Tpt	Tpt	Tpt	Tpt
**LTU**	Cst	Cst	Pub	Cst	Cst	Fod	Fod	Cst	Cst	Cst	Cst	Cst	Cst	Cst	Cst	Fod	Fod
**LUX**	Fin	Fin	Fin	Fin	Fin	Fin	Fin	Fin	Fin	Fin	Fin	Fin	Fin	Fin	Fin	Fin	Fin
**LVA**	Agr	Otr	Fod	Fod	Cst	Cst	Cst	Cst	Cst	Cst	Cst	Cst	Cst	Cst	Cst	Cst	Cst
**MEX**	Fod	Fod	Cst	Cst	Fod	Cst	Cst	Cst	Cst	Cst	Cst	Cst	Cst	Cst	Cst	Cst	Cst
**MLT**	Fod	Fod	Fod	Fod	Fod	Fod	Fod	Fod	Fod	Fod	Ocm	Ocm	Ocm	Ocm	Ocm	Ocm	Ocm
**NLD**	Fod	Cst	Fod	Cst	Cst	Cst	Cst	Cst	Cst	Cst	Cst	Cst	Cst	Cst	Cst	Fod	Fod
**POL**	Fod	Fod	Fod	Fod	Fod	Cst	Fod	Fod	Fod	Fod	Fod	Fod	Fod	Cst	Cst	Cst	Cst
**PRT**	Cst	Cst	Cst	Cst	Cst	Cst	Cst	Cst	Cst	Cst	Cst	Cst	Cst	Cst	Hth	Hth	Hth
**ROM**	Sal	Fod	Sal	Sal	Sal	Sal	Sal	Sal	Fod	Cst	Fod	Cst	Cst	Cst	Cst	Cst	Cst
**RUS**	Hth	Hth	Hth	Hth	Fod	Hth	Hth	Hth	Hth	Hth	Hth	Hth	Hth	Hth	Hth	Hth	Hth
**SVK**	Pub	Pub	Cst	Pub	Pub	Cst	Ele	Ele	Ele	Cst	Cst	Cst	Cst	Cst	Cst	Cst	Cst
**SVN**	Cst	Cst	Cst	Cst	Cst	Cst	Cst	Cst	Cst	Cst	Cst	Cst	Cst	Cst	Cst	Cst	Cst
**SWE**	Hth	Obs	Obs	Hth	Obs	Obs	Obs	Tpt	Tpt	Tpt	Tpt	Tpt	Tpt	Tpt	Obs	Tpt	Tpt
**TUR**	Tex	Tex	Fod	Fod	Tex	Tex	Tex	Tex	Tex	Tex	Tex	Tex	Tex	Tex	Tex	Tex	Tex
**TWN**	Fod	Pub	Pub	Pub	Elc	Elc	Elc	Elc	Elc	Elc	Elc	Elc	Elc	Elc	Elc	Elc	Elc
**USA**	Pub	Pub	Pub	Pub	Pub	Pub	Pub	Hth	Hth	Hth	Hth	Hth	Pub	Pub	Pub	Pub	Pub
