# Supplementary material for: World Input-Output Network
Source: PLoS One. 2015 Jul 29;10(7):e0134025. doi: 10.1371/journal.pone.0134025 (PMC4519177; doi:10.1371/journal.pone.0134025)
Supplement: S9 Table — The codes of countries and industries can be found in S1 Table and S2 Table. (PDF) [file pone.0134025.s010.pdf]

Industry/Year	1995	1996	1997	1998	1999	2000	2001	2002	2003	2004	2005	2006	2007	2008	2009	2010	2011
**Agr**	USA	USA	USA	USA	CHN	USA	CHN	CHN	CHN	CHN	USA	CHN	CHN	CHN	CHN	CHN	CHN
**Min**	USA	USA	USA	CHN	CHN	USA	USA	USA	CHN	CHN	USA	CHN	CHN	CHN	CHN	CHN	CHN
**Fod**	JPN	USA	USA	USA	JPN	USA	USA	USA	CHN	CHN	CHN	CHN	CHN	CHN	CHN	CHN	CHN
**Tex**	CHN	CHN	CHN	CHN	CHN	CHN	CHN	CHN	CHN	CHN	BRA	CHN	CHN	CHN	CHN	CHN	CHN
**Lth**	CHN	CHN	CHN	CHN	CHN	CHN	CHN	CHN	CHN	CHN	BRA	CHN	CHN	CHN	CHN	CHN	CHN
**Wod**	JPN	JPN	JPN	JPN	JPN	JPN	JPN	JPN	JPN	JPN	CHN	CHN	CHN	CHN	CHN	CHN	CHN
**Pup**	USA	USA	USA	JPN	JPN	JPN	JPN	JPN	JPN	JPN	USA	USA	USA	USA	USA	USA	USA
**Cok**	USA	USA	USA	JPN	USA	USA	USA	CHN	USA	CHN	USA	USA	USA	USA	CHN	USA	USA
**Chm**	JPN	JPN	JPN	JPN	JPN	JPN	JPN	JPN	CHN	CHN	CHN	CHN	CHN	CHN	CHN	CHN	CHN
**Rub**	USA	USA	USA	USA	USA	USA	USA	USA	USA	USA	USA	USA	CHN	CHN	CHN	CHN	CHN
**Omn**	CHN	CHN	CHN	CHN	CHN	CHN	CHN	CHN	CHN	CHN	CHN	CHN	CHN	CHN	CHN	CHN	CHN
**Met**	JPN	JPN	JPN	USA	USA	USA	USA	USA	CHN	CHN	CHN	CHN	CHN	CHN	CHN	CHN	CHN
**Mch**	USA	USA	USA	USA	USA	USA	USA	CHN	CHN	CHN	CHN	CHN	CHN	CHN	CHN	CHN	CHN
**Elc**	USA	USA	USA	USA	USA	CHN	CHN	CHN	CHN	CHN	CHN	CHN	CHN	CHN	CHN	CHN	CHN
**Tpt**	USA	USA	USA	USA	USA	USA	USA	USA	USA	USA	USA	USA	USA	CHN	CHN	CHN	CHN
**Mnf**	USA	USA	USA	USA	USA	USA	USA	USA	USA	USA	USA	USA	USA	USA	USA	USA	USA
**Ele**	JPN	USA	USA	USA	USA	USA	USA	USA	JPN	USA	USA	USA	USA	USA	JPN	JPN	JPN
**Cst**	JPN	JPN	JPN	JPN	JPN	JPN	JPN	CHN	CHN	CHN	USA	USA	CHN	CHN	CHN	CHN	CHN
**Sal**	JPN	JPN	JPN	JPN	JPN	JPN	JPN	JPN	JPN	JPN	JPN	JPN	JPN	JPN	JPN	JPN	JPN
**Whl**	JPN	USA	USA	USA	USA	USA	USA	USA	USA	USA	USA	USA	USA	USA	USA	USA	USA
**Rtl**	USA	USA	USA	USA	USA	USA	USA	USA	USA	USA	USA	USA	USA	USA	USA	USA	USA
**Htl**	JPN	JPN	JPN	JPN	JPN	JPN	JPN	JPN	JPN	JPN	JPN	JPN	JPN	JPN	JPN	JPN	JPN
**Ldt**	JPN	JPN	JPN	JPN	JPN	JPN	JPN	JPN	JPN	JPN	USA	USA	USA	USA	IND	IND	IND
**Wtt**	USA	USA	CHN	CHN	CHN	CHN	CHN	CHN	CHN	CHN	BGR	CHN	CHN	CHN	CHN	CHN	CHN
**Ait**	USA	USA	USA	USA	USA	USA	USA	USA	USA	USA	USA	USA	USA	USA	USA	USA	USA
**Otr**	USA	USA	USA	USA	USA	USA	USA	USA	USA	USA	BEL	USA	USA	USA	USA	USA	USA
**Pst**	USA	USA	USA	USA	USA	USA	USA	USA	USA	USA	USA	USA	USA	USA	USA	USA	USA
**Fin**	JPN	JPN	JPN	USA	USA	USA	USA	USA	USA	USA	USA	USA	USA	USA	USA	USA	USA
**Est**	USA	USA	USA	USA	USA	USA	USA	USA	USA	USA	USA	USA	USA	USA	USA	USA	USA
**Obs**	USA	USA	USA	USA	USA	USA	USA	USA	USA	USA	USA	USA	USA	USA	USA	USA	USA
**Pub**	USA	USA	USA	USA	USA	USA	USA	USA	USA	USA	USA	USA	USA	USA	USA	USA	USA
**Edu**	JPN	JPN	JPN	JPN	JPN	USA	USA	USA	USA	CHN	CHN	CHN	CHN	CHN	CHN	CHN	CHN
**Hth**	USA	USA	USA	USA	USA	USA	USA	USA	USA	USA	USA	USA	USA	USA	USA	USA	USA
**Ocm**	JPN	USA	USA	USA	USA	USA	USA	USA	USA	USA	USA	USA	USA	USA	USA	USA	USA
**Pvt**	USA	USA	USA	USA	USA	USA	USA	USA	USA	USA	BGR	USA	USA	USA	USA	USA	USA
